# Mechanical characterization of squid giant axon membrane sheath and influence of the collagenous endoneurium on its properties

**DOI:** 10.1038/s41598-019-45446-y

**Published:** 2019-06-20

**Authors:** Annaclaudia Montanino, Astrid Deryckere, Nele Famaey, Eve Seuntjens, Svein Kleiven

**Affiliations:** 1 Division of Neuronic Engineering, Royal Institute of Technology (KTH), Huddinge, Sweden; 2 Laboratory of Developmental Neurobiology, Department of Biology, KU Leuven, Leuven, Belgium; 3 Biomechanics section, KU Leuven, Leuven, Belgium

**Keywords:** Cell death in the nervous system, Mechanical engineering, Biomaterials

## Abstract

To understand traumas to the nervous system, the relation between mechanical load and functional impairment needs to be explained. Cellular-level computational models are being used to capture the mechanism behind mechanically-induced injuries and possibly predict these events. However, uncertainties in the material properties used in computational models undermine the validity of their predictions. For this reason, in this study the squid giant axon was used as a model to provide a description of the axonal mechanical behavior in a large strain and high strain rate regime $({\boldsymbol{\varepsilon }}={\bf{10}}{\boldsymbol{ \% }},\,\mathop{{\boldsymbol{\varepsilon }}}\limits^{\cdot }={\bf{1}}{{\boldsymbol{s}}}^{-{\bf{1}}})$, which is relevant for injury investigations. More importantly, squid giant axon membrane sheaths were isolated and tested under dynamic uniaxial tension and relaxation. From the lumen outward, the membrane sheath presents: an axolemma, a layer of Schwann cells followed by the basement membrane and a prominent layer of loose connective tissue consisting of fibroblasts and collagen. Our results highlight the load-bearing role of this enwrapping structure and provide a constitutive description that could in turn be used in computational models. Furthermore, tests performed on collagen-depleted membrane sheaths reveal both the substantial contribution of the endoneurium to the total sheath’s response and an interesting increase in material nonlinearity when the collagen in this connective layer is digested. All in all, our results provide useful insights for modelling the axonal mechanical response and in turn will lead to a better understanding of the relationship between mechanical insult and electrophysiological outcome.

## Introduction

Traumatic injury to the human central nervous system (CNS) and peripheral nervous system (PNS) arises as a result of the application of high dynamic loads to the head or the spinal cord and the limbs, respectively. Due to the mechanical insult, the nervous tissue undergoes non-physiological deformations that can entail morphological alterations at the cellular and subcellular level^1–5^. Depending on the injury severity, these morphological alterations could lead to axonal degeneration and death. Even in non transected axons, as a consequence of the mechanical deformation, an alteration in the neural tissue electro-physiological response can be observed^6–10^. Interestingly, even in non-injurious scenarios, axons present coupled electrical, thermal, and mechanical phenomena^11^–such as the mechanical surface waves that accompany action potential propagation^12–15^. In the axon, therefore, the mechanical and electrical response are intertwined.

To gain understanding of the axonal injury mechanism and to establish injury thresholds (both morphological and functional ones), researchers have relied on experimental models. Induced morphological and electrophysiological changes have been investigated at the tissue-level, for example, using an *in vivo* guinea pig optic nerve stretch model^8,16^ or *ex vivo* guinea pig spinal cords strips^17–19^. Additionally, *in vitro* models, such as organotypic and dissociated primary cultures, have been extensively used to examine the effect of mechanical perturbations on networks of unmyelinated axons (see^20^ for an extensive review). Using these models, the compound response of myelinated or unmyelinated axons can be studied. However, inferences about the viability of individual axons cannot be made.

With the aim of analyzing the mechanical-electrical response of a single axon (rather than the cumulative response of several ones belonging to a nerve bundle), a seminal study was conducted on the squid giant axon^6,21^. In that study, squid giant axons (SGAs) were stretched at low strain rate ($\dot{\varepsilon }\approx 0.003 \mbox{-} 0.3{s}^{-1}$) to reveal a mechanical threshold for electrical impairment. In particular, the mechanical behavior of the whole SGA was reported along with the post-stretch action potentials. The SGA is an unmyelinated axon, whose bioelectric properties were first discovered by Young *et al*.^22,23^. Although this neurite’s diameter is considerably bigger than that of human or any other mammalian axons, it has proved a invaluable model to explain neurons’ electrophysiology^24^.

Unlike unmyelinated axons in the mammalian CNS, the SGA membrane sheath presents additional layers on top of the lipid-bilayer. Similarly to small axons in the PNS^25^, in fact, the SGA is ensheated by a layer of non-myelinating Schwann cells and, always in analogy with peripheral nerve fibers, this axon is also enwrapped in a collagenous layer: the endoneurium^26^. In humans affected by traumatic injury to peripheral nerves, this layer seems to be of vital importance^27^. Following axonotmesis^28^, in fact, even when axons and myelin sheaths are broken and undergo Wallerian degeneration, an intact or partially intact endoneurium may yield axonal regrowth. Knowing the mechanical response of this structure was also reported to be compelling for the development of effective surgical nerve repair strategies^29^.

A need for mechanical properties of the axons, and especially of its membrane sheath, was also expressed by the axonal modelling community. Recently, in fact, the connection between mechanical and electrophysiological axonal activity has been investigated with computational models. The relationship between longitudinal axonal strain and action potential has been modelled in 1D^30^. In that study, motivated by the lack of membrane focused data, the authors assumed axonal incompressibility and a one-to-one stretch behavior between the membrane and axoplasm to analytically compute the surface membrane strain from the microscopic axial strain. In another study, altered axonal morphologies were shown to affect conduction velocity and firing frequency^31^. However, in absence of a material description, the geometrical changes were given as an input rather than being the result of a mechanical load. In a more recent study instead, the axolemma was explicitly modelled and embedded in a 3D electro-mechanical finite element model of a single neuron^32^. Such model was validated, among others, against SGA data. Moreover, the researchers highlighted the need of axon-specific mechanical data (namely, tissue properties) to obtain accurate results from the coupled electro-mechanical model. Stresses in the axonal membrane, which were found to be very dependent on the assigned material properties, were considered to significantly affect the electrical response. Finally, although unrelated to axonal injury, the need of SGA’s sheath properties was also made explicit in a study predicting axonal membrane displacement driven by the propagation of the action potential^33^.

The above suggests that accurate mechanical modelling of the axon and its membrane sheath is necessary to predict not only the mechanical, but also the physiological axonal activity. The SGA is a model that has been vastly used to understand axonal electrophysiology even under mechanical perturbation. However, the mechanical properties of its membrane sheath have not been previously investigated. The focus of this work is therefore a mechanical characterization of the SGA and, in particular, of its membrane sheath at large strains and high strain rate. Furthermore, the influence of the collagen-rich endoneurium on the total membrane sheath response was assessed.

## Materials and Methods

### Sample preparation

**Figure 1:**
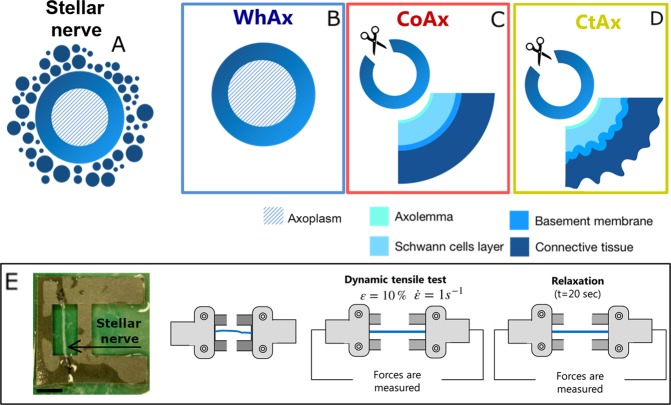
Schematic representation of sample preparation and testing. (**A**) Stellar nerve section as it appears when dissected from the animal. (**B**) Whole axon (WhAx) obtained by fine cleaning of the stellar nerve. (**C**) Cut open axon (CoAx) obtained from whole axon through slitting procedure. The composition of the membrane sheath is also depicted. (**D**) Collagenase treated axon (CtAx) obtained from CoAx samples by treating them with Collagenase/Dispase. An altered morphology of the endoneurium is also depicted. (**E**) Sample mounted on a sand-paper slit that is then placed in the testing machine for the initial dynamic tensile test, followed by 20 seconds of relaxation. Scale bar represents 1 cm.

Squids (*Loligo vulgaris*) were purchased at a local fish store where they were kept (already deceased) at a temperature of 2 °C. The animals, destined for the food market, were caught 1 day earlier in the North sea and tested within 10 hours from purchase. The dissection of the giant axons was performed according to guidelines published in Adelman *et al*.^34^. Briefly, once the largest stellar nerve was identified, this was delicately isolated from the mantle tissue starting from the stellate ganglion up to the main bifurcation. The two nerve ends were then tied with cotton threads to prevent axoplasm outflow. The nerve was then cut and moved to a Petri dish with artificial calcium-free sea water (450 mM NaCl, 10 mM KCl, 50 mM MgC*l*_2_, 10 mM HEPES, 3 mM EGTA, pH = 7.6–7.8). From each specimen, two samples, a proximal and a distal one, were isolated. Each one was mounted on a sandpaper slit (Fig. 1E) with a central 2 cm long-window and placed in a Sylgard-coated Petri dish (Living System Instrumentation) submerged in calcium-free sea water and kept in the refrigerator at 5 °C until the fine cleaning procedure.

**Figure 2:**
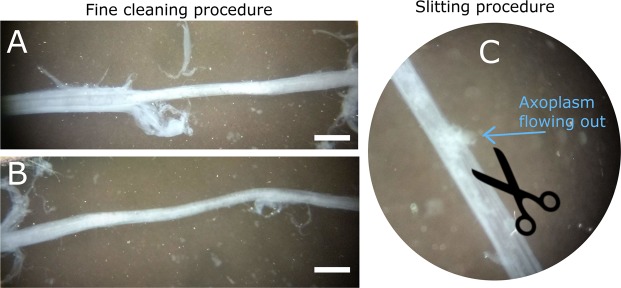
(**A**) During fine cleaning, small nerve fibers are removed from the sides of the squid giant axon. (**B**) Fine-cleaned squid giant axon. (**C**) Example of slitting procedure showing half cut-open portion of the squid giant axon with axoplasm flowing out. Scale bar represents 600 *μ*m.

To fine-clean the stellar nerve and isolate the giant axon, the sample was placed under a dissection microscope (ZEISS, Stemi DV4). During this procedure, the small nerve fibers surrounding the giant axon were held with forceps and teased apart with microscissors (Vannas Spring Scissors −2 mm Cutting Edge, Fine Science Tools) (Fig. 2A,B). Only the giant axons that did not show any damage (perforation with evident outflow of axoplasm and consequent opaque and constricted regions) were considered for the following mechanical tests. Prior to testing, each sample was imaged with a digital microscope DinoLite premier (AnMo Electronics Corp., Hsinchu, Taiwan) to measure its diameter.

At this stage, the generic giant axon sample can be described as a tube with an inner fluid-like compartment, the axoplasm (Fig. 1B). The tube walls are instead a composite membrane sheath. From the lumen outward, the sheath presents, the axolemma (plasma membrane), a layer of Schwann cells followed by the basement membrane and a prominent layer of loose connective tissue consisting of fibroblasts and collagen^35^. In the remainder of the manuscript, three types of samples will be considered: whole giant axons (WhAx, Fig. 1B), cut-open axons (CoAx, Fig. 1C) and collagenase-treated axons (CtAx, Fig. 1D). CoAx samples were obtained through slitting procedure. Specifically, giant axons were cut-open using the microscissors tip (Fig. 2C). Once the axon was cut-open, most of the axoplasm flowed out and the rest was gently washed away with a pipette. Finally, CtAx samples were obtained from cut-open axons that were then mounted on sand-paper slits and submerged for 30 min in a solution of Ca-free ASW and Collagenase/Dispase (1 mg/mL, Roche) in a water bath at T = 37 °C. The samples were then rinsed in Ca-free ASW and kept refrigerated until testing.

### Uniaxial tensile test and relaxation

The samples were mounted using sandpaper slits onto the tension grips of a LM1 Test Bench (BOSE) equipped with a 250 grams (2.55 N) Load cell (Fig. 1E). Once mounted, the supporting lateral sandpaper stripes were cut and the sample was slightly manually pretensioned (*F*_0_ = 0.0029 ± 0.0025) to eliminate possible slack in the sample. Each specimen was then subjected to a dynamic displacement-controlled tension (up to 10% strain, strain rate 1 *s*^−1^). This rate is representative of an injury scenario, while the deformation was chosen purposefully to be safely below 20%, which was shown to be an irreversible functional injury threshold specifically for the squid giant axon^6^. The strain was then kept constant for 20 seconds to perform a relaxation test. Force data were acquired at 4000 Hz sampling rate in the elastic loading phase and at 25 Hz in the late relaxation phase (t > 1 sec). The same test was performed on WhAx, CoAx and CtAx samples.

### Contribution of the membrane sheath to the mechanical behavior of the whole giant axon

First, a comparison between the whole giant axon (WhAx) and the cut-open axon (CoAx) (Fig. 1B,C) was conducted, the aim being assessing the mechanical contribution of the membrane sheath to the mechanical behavior of the whole giant axon. To this end, while 10 samples were tested directly after undergoing fine-cleaning, 14 other samples underwent the slitting procedure^34^ prior to testing.

Not knowing a priori what is to be considered the load bearing structure in the WhAx samples (and hence what area needs to be considered in the computation of stresses), force-displacement (F-x) and force-time (F-t) responses were considered to compare WhAx against CoAx samples. The force-displacement responses were fitted with a nonlinear spring model, *F* = *F*_0_ + *k*_1_*x* + *k*_2_*x*^3^, where *F*_0_ is the force applied to slightly pre-tension the sample and *k*_1_, *k*_2_ are the linear and nonlinear coefficients respectively. These coefficients where then used to statistically compare the tensile response of the two sample sets. Moreover, to compare their relaxation behavior, the normalized percentual relaxation was derived as f^*^ = (*F*_*in*_ − *F*_*fin*_)./*F*_*in*_, where *F*_*in*_ is the initial force and *F*_*fin*_ is the relaxed force after 20 seconds.

### Mechanical characterization of the membrane sheath

To describe the samples’ behavior under tension, the first Piola-Kirchhoff stress in the direction of the applied deformation *P*_*xx*_ was computed from the measured force *F*, by dividing the latter by the samples initial cross-sectional area. For CoAx and CtAx samples, the cross-sectional area was computed as the area of an annulus with outer radius *R* equal to the original sample radius (prior to slitting procedure) and inner radius *r* equal to *R* − 7 *μm*, where 7 *μm* is the reported average thickness of the membrane sheath in a 500-*μm*-diameter *Loligo paelei* giant axon^35^. At this stage, only the elastic/hyperelastic response was considered, neglecting the time-dependency. Hence, the tensile curves were fitted using a one-term Ogden constitutive model^36^ with strain energy function $W({\lambda }_{x},{\lambda }_{y},{\lambda }_{z})=\mu ({\lambda }_{x}^{\alpha }+{\lambda }_{y}^{\alpha }+{\lambda }_{z}^{\alpha }-3)$, where *μ* and *α* are material constants that need to satisfy *μα* = 2***μ***, where ***μ*** is the classic shear modulus in the original configuration. Let *λ*_*x*_ = *λ* be the stretch in the axial direction calculated as $\lambda =\frac{L+x}{L}$, where *L* is the initial sample length measured as the clamps distance and *x* is the applied displacement. Assuming the material to be incompressible (*λ*_*x*_*λ*_*y*_*λ*_*z*_ = 1) the stretches in the orthogonal directions can be expressed as *λ*_*z*_ = *λ*_*y*_ = $\frac{1}{\sqrt{\lambda }}$. Under uniaxial tension (*P*_*zz*_ = *P*_*yy*_ = 0), the following constitutive expression holds for the stress in the axial direction: ${P}_{xx}={P}_{0}+\mu ({\lambda }_{x}^{(\alpha -1)}-{\lambda }_{x}^{(\frac{-\alpha }{2}-1)})$, where *P*_0_ was included in the fit to take into account the applied pre-tension when fitting the experimental curves.

The curves were fitted using two different approaches: the naive average approach (NA) and the standard two stages (STS)- traditionally used with pharmacokinetic data^37^. With the first approach, all sample curves are averaged and the model is fitted (using a nonlinear least squares algorithm) to the average curve. With the STS approach instead, each sample curve is individually fitted with the model of choice and the tissue parameters *θ* are computed as the mean of the individual samples parameters.

The relaxation function *G*(*t*) was obtained by dividing the stress *P*_*xx*_(*t*) by the applied initial nominal strain (*E*(*t*) = *P*_*xx*_(*t*)/*ε*_0_) and using the well known relation between Young’s modulus and shear modulus (*E* = *G*/2(1 + *ν*)). The normalized shear stress *g*_*R*_(*t*) = *G*(*t*)/*G*(0) was fitted with a Prony series with 1, 2, 3 terms:

$${g}_{R}(t)=1-{\sum }_{i=1}^{terms}{g}_{i}(1-{e}^{-t/{\tau }_{i}})\infty $$

### Influence of collagen on the membrane sheath response

**Figure 3:**
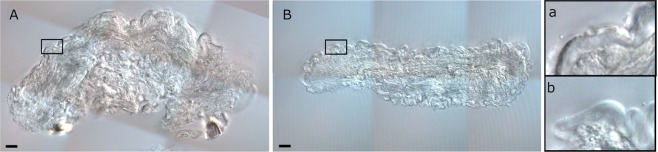
(**A**) Differential interference constrast microscopy image of a control sample showing a thick and dense connective tissue layer, the endoneurium. Rectangle zoomed in (a). (**B**) After collagenase treatment, the CtAx sample shows a relaxed and wavy connective layer. Rectangle zoomed in (b). Scale bars represent 10 *μ*m.

Finally, we assessed the influence of collagen on the mechanical response of the membrane sheath. Axons were fixed in 4% paraformaldehyde in phosphate-buffered saline (PBS) overnight at 4 °*C*. Afterwards, they were washed in PBS and embedded in 4% agarose (Invitrogen). 100 *μ*m-thick cross- sections were made using a vibratome. Sections were imaged on a Leica DM6 B upright microscope. Imaging of membrane sheath cross-sections of both control and collagenase-treated axons (CtAx) was performed to confirm the effect of the treatment. Figure 3 shows a degradation of collagen and the consequent relaxation of the connective sheath.

To be able to statistically compare the tensile response of CoAx and CtAx samples, the classic shear modulus ${\boldsymbol{\mu }}=\frac{\mu \alpha }{2}$ and the nonlinear parameter *α* were derived from each sample curve and the distributions of these parameters for the two sample sets were analyzed with an unpaired *t-*test. To assess the time-dependent behavior, the normalized relaxation percentage at 20 seconds (${g}^{\ast }(20\,sec)$) was chosen as a means of comparison.

## Results

### Contribution of the membrane sheath to the mechanical behavior of the whole giant axon

**Figure 4:**
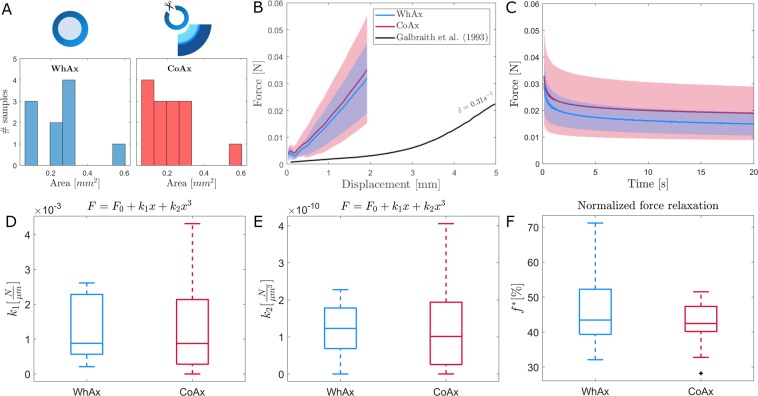
(**A**) Cross sectional area distributions of the tested whole axon samples WhAx (left) and the cut-open samples CoAx (right). (**B**,**C**) Force-displacement and force relaxation response of WhAx and CoAx samples presented as means (solid lines) and standard deviations (shaded areas). (**D**–**F**) Boxplots of parameters *k*_1_, *k*_2_ and *f*^   *^ for WhAx and CoAx samples.

The first aim of the study was to assess the mechanical contribution of the squid giant axon membrane sheath to the response of the whole axon. Figure 4 shows the comparison between samples belonging to the WhAx and the CoAx sample sets. The samples’ original cross sectional area are reported as column plots in Fig. 4A. These distributions were then compared using Wilcoxon rank-sum test, which showed no significant difference, i.e. samples can be assumed to belong to distributions with equal medians.

Figure 4B shows the average mechanical response in tension of WhAx (N = 10) and CoAx samples (N = 14) and their respective standard deviations. In Fig. 4C the mean force relaxation curves and the standard deviation for both samples sets are reported. Figure 4D and Fig. 4E show the boxplots of the two parameters *k*_1_ and *k*_2_ that identify the tensile response. The low order of magnitude of parameter *k*_2_ shows that the tensile response is close to linear in the tested deformation range. This can also be observed in the plot in Fig. 4B. The boxplot in Fig. 4F shows the percentual normalized force relaxation *f*^   *^ for both sample sets. Given the similarity between the mechanical responses of these two sample sets, the burden of proof was shifted towards proving equivalence rather than difference. Hence, a *two-one-sided t-tests* (TOST)^38^ was used, the null hypothesis being that there is a true effect larger in absolute value than the Smallest Effect Size of Interest (SESOI). For our three parameters *k*_1_, *k*_2_ and *f*^   *^ the SESOI was chosen as 0.001 *N*/*µm*, 1 × 10^−10^ N/m^3^, and 10% respectively. These tests revealed that for both *k*_1_ and *k*_2_ the 95% confidence interval (CI) around the effect falls completely within the equivalence range, meaning that equivalence can be claimed within this range. On the contrary *f*^   *^ distributions did not pass the test, hence equivalence cannot be claimed.

### Mechanical behavior of squid giant axon membrane sheath

**Figure 5:**
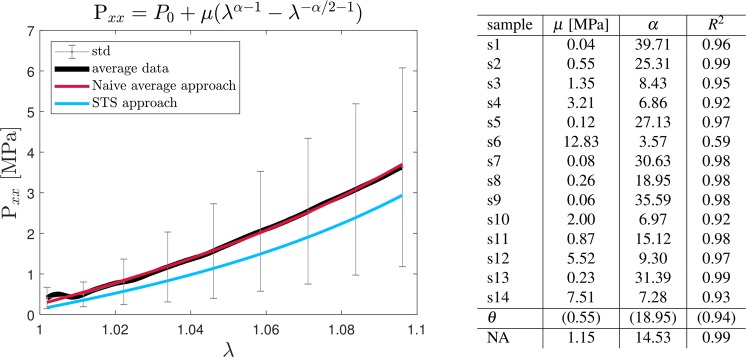
*Left:* Stress versus stretch behavior of CoAx samples. The average response derived with naive average approach and standard two stage approach are reported in red and blue respectively. *Right:* Constitutive parameters with the corresponding coefficient of determination *R*^2^ obtained from calibrating each sample curve with a one term Ogden model. STS-obtained and NA-obtained tissue parameters are also reported.

**Figure 6:**
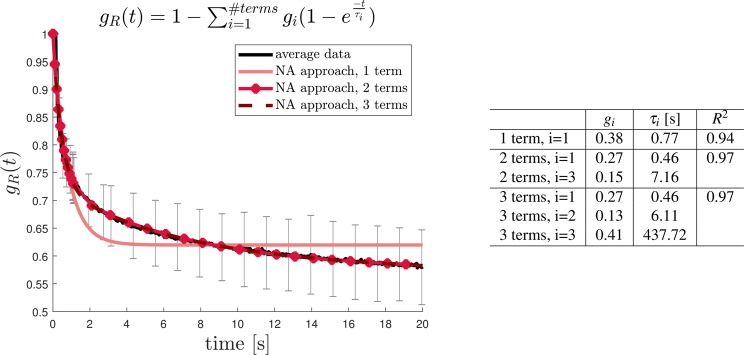
*Left:* Normalized shear relaxation modulus of CoAx samples. The average response (black) was fitted with naive average approach and standard two stage approach. The resultant fit curves are plotted in red and blue respectively. *Right:* Material parameters from the Prony series expansion with 1, 2 or 3 terms.

The second aim of the study was to characterize the mechanical behavior of the squid giant axon membrane sheath in tension and relaxation. The average experimental response of CoAx samples was fitted with a one term-Ogden model and so was each individual curve. The average response shows only a slight nonlinear behavior up to the tested stretch $\lambda \approx 10 \%$. Both fitting approaches (NA and STS) reflect this behavior. The table in Fig. 5 (*right*) summarizes the material parameters obtained when fitting all samples individually and the population parameters obtained with either the STS or NA approach. Figure 6 shows the mean normalized relaxation modulus as well as the fit with Prony series with 1, 2 or 3 Maxwell branches. Both plot and table in Fig. 6 indicate that at least two branches are needed to describe the relaxation behavior of the samples and that adding a third branch does not increase the accuracy of the fit since the coefficient of determination remains *R*^2^ = 0.97.

### Influence of collagen on the membrane sheath response

**Figure 7:**
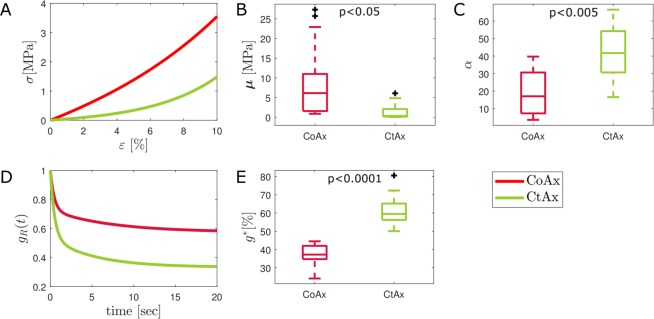
Comparison between CoAx (N = 14) and CtAx samples (N = 9). (**A**) Average tensile response of the two sets of samples. (**B**) Boxplot showing the distribution of the classic shear modulus for each of the sets. (**C**) Boxplot showing the distribution of the nonlinear parameter *α* for each set. (**D**) Average normalized shear modulus for the two sample sets. (**E**) Boxplot showing the distribution of the parameter g^*^(20 *sec*) that is the percentual normalized relaxation.

Figure 7 displays a comparison between the mechanical response of CoAx samples and the CtAx ones. The average responses (obtained with NA approach) in Fig. 7A show a softer behavior of the collagenase-treated samples. Figure 7B is a boxplot of the classic shear modulus for the two analyzed types of samples. The unpaired *t-*test performed on this parameter evidenced a statistical difference between the two distributions (p < 0.05). When adjusting the significance level for multiple comparisons (two in our case), however, this parameter did not survive Bonferroni correction. The distributions of parameter *α* (Fig. 7C) were also found to be different at the unpaired *t-*test (p < 0.005). Furthermore, in Fig. 7D the differences between the normalized relaxation response of the two samples average can be appreciated. Samples that had been depleted of collagen show an increased fluid-like behavior and this is confirmed by the unpaired *t-*test performed on g^*^(20 *sec*) (p < 0.0001).

## Discussion

Research into the mechanics of single neurons and neuron bundles is vital for a better understanding of axonal injury mechanisms. To try and capture the intertwined mechanical and electrical nature of the axon, computational models rely on an accurate choice of parameters to achieve reliable predictions. In particular, the axonal membrane has a pivotal role in the conduction of the electric signal and its mechanical properties affect the prediction of computational models’ electrical output^32^. So far, its specific mechanical properties have been neglected and assumed to be similar to those of any plasma membrane. This similarity does not hold, especially in the case of the squid giant axon, due to the composite nature of this structure. The novel contribution of this study lies in the mechanical characterization of the isolated squid giant axon’s membrane sheath under large strain and at high strain rate. For the first time, the contribution of the squid giant axon membrane sheath to the whole axon response was assessed and the material constitutive properties for this enwrapping layer were provided.

Single axons tested in previous studies have shown an overall linear behavior under tension^39,40^. In a microneedle experiment by Bernal *et al*.^41^, the stiffness of single axons was probed by displacing the midpoint of each neurite, of generic initial length *L*_*x*_, perpendicularly to the neurite’s axis, yielding a final neurite length ${L}_{0}={L}_{x}/cos(\theta )$ (see Fig. 3 in the original publication for geometrical clarification). Data from this experiment were expressed by means of force-angle (F-*θ*) curves, which showed a pronounced nonlinear behavior. Noting that nominal strains along the axon in this case are ${\varepsilon }_{ax}=(\frac{1}{cos\theta }-1)$, one can plot the same data as force-deformation (F-${\varepsilon }_{ax}$) curves, which show a lesser nonlinear behavior. In a previous study focusing on squid giant axon mechanics^6^, a nonlinear force-displacement relationship was evidenced. However, it must be noted that the presented curves span strains $\varepsilon =0 \mbox{-} 30 \%$ (calculated from the reported length to diameter ratio of 30 and the average sample diameter of 500 *μm*) and indeed shows a nonlinear increase of the force around $\varepsilon =20 \%$, which is twice the maximum strain used in this study. Compared to the latter study, the forces measured in our study are an order of magnitude higher, probably due to the difference in strain rate ($\approx 0.3{s}^{-1}$ in^6^) or the different specimen conditions. All in all, our results on whole axons are in line with the literature and hint that, within the considered deformation range ($\varepsilon \in [0 \mbox{-} 10] \%$), nonlinearity sources in the brain tissue might need to be found at another scale or at least in other brain constituents rather than the neurons.

Understanding the distribution of loads within different peripheral nerve structures has been the focus of previous studies^42–45^. However, at the neuronal and subneuronal level, the understanding of load distributions is hindered by both the size of mammalian neurons and the close relation between the axonal membrane and the axonal cytoskeleton^46^. Using the squid giant axon as a model comes with the possibility of separately analyzing its constituents. In the current study, the membrane sheath was isolated through a slitting procedure. When assessing the differences between WhAx and CoAx samples, forces-displacement (F-x) responses were considered. Although the slitting procedure could induce the release of potential residual stresses, hindering this way the comparison between WhAx and CoAx configuration, we believe that such a procedure would mostly affect the circumferential forces rather than the longitudinal ones, which are the object of our attention. Additionally, considering the quasi-linearity of our samples, computing an elastic modulus considering or disregarding residual stresses would most likely yield similar results. Moreover, given the small intracellular pressures that were measured in the squid giant axon (~mPa^47^), it can be speculated that, if any, residual stresses would be several orders lower than those induced by our deformations. As a result of our comparison, statistical equivalence was observed between the elastic parameters (*k*_1_, *k*_2_) of whole axons and that of isolated membrane sheath samples, indicating that, when a whole squid giant axon is stretched, the structure that is actually bearing the load is the membrane sheath. Indeed, previous studies have shown that the squid axoplasm - isolated from the membrane sheath- presents a low resistance to stretch^48^. When deformed with $\varepsilon =100 \%$, in fact, the measured forces were in the order of 100 *μN*, which are several orders of magnitude smaller than those measured in the current study.

Overall, the membrane sheath shows the characteristics of a viscoelastic material. The one-term Ogden model proved sufficient to explain the experimental tensile curves. In our results, a large inter-sample variability is apparent. This is to some extent inherent in biological materials, but in our case it could also have been enhanced by the cleaning procedure, which is done on each of the samples, or the different animals’ size. Finally, the lack of thickness information, which is addressed in the following section, might also inflate the standard deviations. Despite the large variability, the mean tensile tissue properties derived with the naive average approach are those of quasi-linear material, which could also justify the usage of the STS approach that should otherwise be avoided for nonlinear materials^49,50^. Given the dynamic load that was applied and the softness of the samples, some inertial effects are visible at low stretches *λ* < 1.01 (Fig. 4B and Fig. 5, left). Nevertheless, the analytical model that is used here is not able to capture such changes – neither we wanted it to–, therefore the large strain description can be considered not to be affected by those initial data points. The stiffness of the membrane sheath was surprisingly high given its translucent and soft appearance. Nevertheless, these properties are in accordance with experiments showing the important role of the endoneurium and its underlying basal lamina in the protection of peripheral nerves at the single fiber level^27,29,45^.

Collagen confers mechanical stability, strength and toughness to a range of tissues. Its arrangement differs depending on the tissue function^51^. Collagen arrangement in the endoneurium of the mouse sciatic nerve was reported to consist of two different layers: in the outer layer, collagen bundles are oriented in the nerve axial direction while the inner layer appears as a delicate interwoven network of thin collagen fibrils^52^. However, in the squid giant axon endoneurium, collagen fibrils cross one another to form a lattice-like arrangement^34^. According to our results, when membrane sheath samples were treated with Collagenase/Dispase, the shear modulus becomes circa 15 times smaller than the original one. In particular the collagen-digested samples have a median stiffness *μ* = 35 kPa, which is approaching the stiffness of cell monolayers^53^. After the treatment, the load is borne by the following structures: the axolemma with residuals of subcortical axoplasm^54^, the Schwann cells layer and the remaining connective tissue fibroblasts. Moreover, it was interesting to observe the increased nonlinearity of the collagenase treated samples. This might be due to the engagement of filament networks connecting the cells or directly to the nonlinearity of the cells’ response^55^. This response was previously overshadowed by the preponderance of the collagen-rich connective tissue.

## Limitations

A major limitation of the study is that the animal samples were dissected several hours *post-mortem* such that no electrical activity can be recorded. Although this would have made the study more complete, the scope of the manuscript was to shed a light on the mechanical properties of the squid giant axon membrane, since the electrical properties have already been studied extensively. Secondly, since measuring sample thickness without fixing the tissue is unfeasible, samples thickness was derived from average data in the literature. Moreover, the membrane sheath mechanical response could include the contribution of the subcortical cytoskeleton, since these two are tightly connected and cannot be easily separated. Although, they might affect the constitutive parameters, these assumptions do not affect the statistical comparisons that were carried out between whole cut-open axons samples and between cut-open and Collagenase-treated ones. Finally, in the future more samples should be considered to be able to draw more statistically relevant conclusions.

## Conclusions

In the present paper, a mechanical characterization of the squid giant axon and its membrane was carried out. In particular, it was shown that when testing the whole giant axon in tension, the sole structure responsible for carrying the load is the membrane sheath. In addition, mechanical properties (elastic and time-dependent) were derived for the membrane sheath that can be used in electro-mechanical models when trying to validate these against experimental data. Finally, the contribution of collagen was shown to be substantial in the membrane sheath response and generic properties for this collagen-depleted tissue were provided.

## Data Availability

The datasets generated during and analysed during the current study are available from the corresponding author on reasonable request.
